# Successful treatment of Miescher's cheilitis in Melkersson-Rosenthal syndrome with betamethasone injections and doxycycline

**Published:** 2012-12-09

**Authors:** Lamia Oudrhiri, Soumiya Chiheb, Farida Marnissi, Soumaya Zamiati, Hakima Benchikhi

**Affiliations:** 1 Department of Dermatology Venerology Ibn Rusd UHC, Casablanca, Morocco; 2 Department of Pathology Ibn Rushd UHC, Casablanca, Morocco

**Keywords:** Miescher's cheilitis granulomatosa, Melkersson-Rosenthal syndrome, treatment

## Abstract

We report a case of a 19-year-old girl who presented with 5-year history of swelling of upper lip and fissured tongue treated with dapsone then oral steroids without any improvement. Clinical examination found peripheral facial nerve paralysis and Labial mucosa biopsy showed non-necrotizing giganto-epithelioid granuloma. Diagnosis of Melkersson-Rosenthal syndrome was retaind because of association of cheilitis, lingua plicata and facial paralysis. Given the failure of dapsone and oral steroid we suggested an association of betamethasone injection and doxycycline. Gradual and permanent reduction of the upper lip volume was observed. One year follow up objectified no reactivation of cheilitis.

## Introduction

Treatment of Miescher's cheilitis granulomatosa integrated or not in Melkersson-Rosenthal syndrome is a real therapeutic challenge. We report a case of 19-year-old girl successfully treated by association of local betamethasone injections and doxycycline.

## Patient and observation

**Figure 1:**
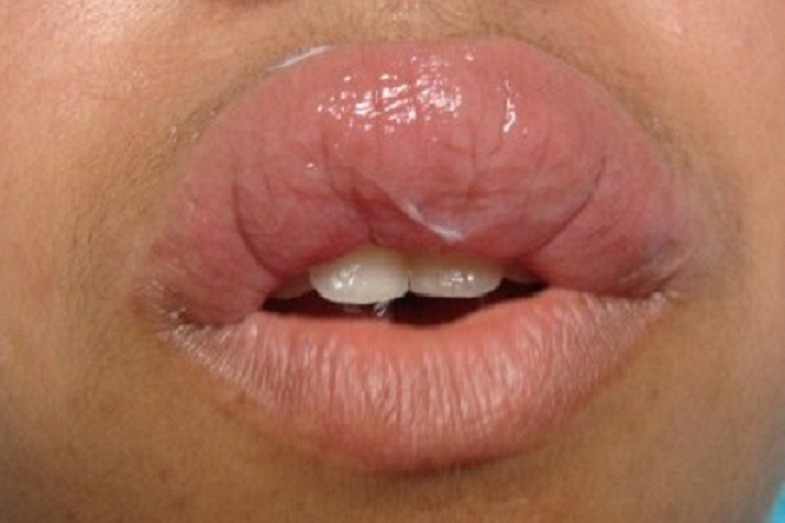
Diffuse swelling of the upper lip.

**Figure 2:**
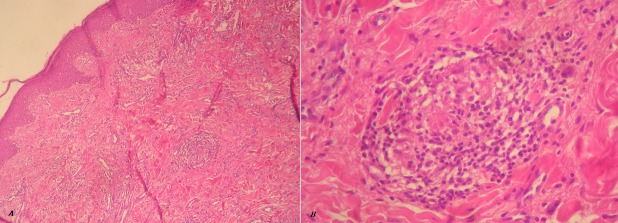
Labial mucosa biopsies showed non-necrotizing giganto-epithelioid granuloma

A 19-year-old girl presented a 5-year history of a painless enlargement of upper lip. Swelling was initially asymmetrical and intermittent then became permanent and diffuse. The patient received dapsone and oral steroids without any improvement. Clinical examination found a diffuse swelling of upper lip (Figure 1) associated to lingua plicata and facial asymmetry. Neurological examination objectified facial nerve palsy. Diagnosis of Melkersson-Rosenthal syndrome was retaind because of association of cheilitis, lingua plicata and facial paralysis. Labial mucosa biopsy showed non-necrotizing giganto-epithelioid granuloma confirming Miescher's cheilitis granulomatosa (Figure 2). A specialized management of caries and dental foci of infection was performed before starting treatment.

**Figure 3:**
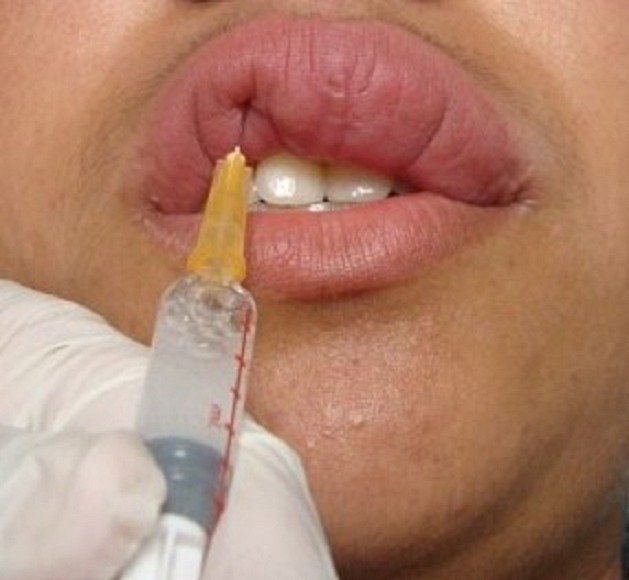
Intralesional betamethasone injection

**Figure 4:**
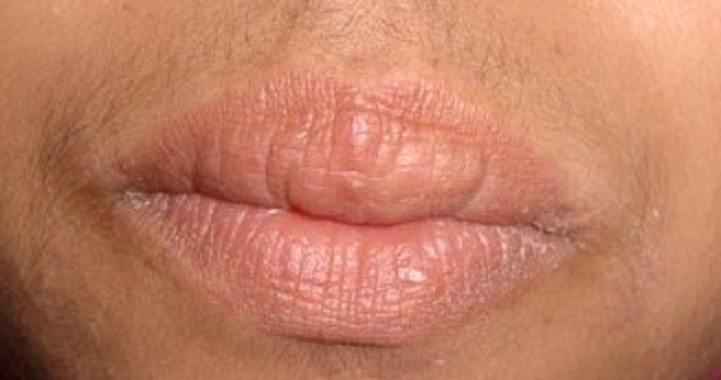
Result after three months of treatment

We prescribed intra-lesional betamethasone injections (7 mg / ml): 1 ml per injection (Figure 3) once a month for three months associated to a single daily dose of doxycycline 200 mg for 3 months. An anesthetic cream with lidocaine and prilocaine (EMLA 5%) was applied one hour before each injection to reduce pain. From the first month of treatment, an important improvement of cheilitis was seen. Gradual and permanent reduction of the upper lip volume was observed after three months of treatment (Figure 4). One year follow up objectified no reactivation of cheilitis.

## Discussion

We report a case of Miescher's cheilitis in Melkersson-Rosenthal syndrome treated successfully by the association betamethasone injections and doxycycline. The use of betamethasone was decided because of non availability of triamcinolone. Intralesional injections of corticosteroids are very painful. A regional anesthesia of a nerve block before administration of triamcinolone may be recommended [1]. In our case local anesthetic cream allowed a good tolerance of treatment.

The management of patients with cheilitis granulomatosa remains a challenge and should be related to the severity of the symptoms.Various therapeutic strategies have been proposed in literature including clofazimine, systemic or intralesional steroids alone or associated to dapsone and in some isolated cases; hydroxychloroquine, metronidazole, thalidomide and infliximab [2–4]. However, treatment is empirical.

The use of cycline is based on its in vitro ability to inhibit granuloma formation by inhibition of protein kinase C. This result provided the successful use of minocycline in the treatment of granulomatous dermatitis [5–7]. The association minocycline and corticosteroids would be more efficient in reducing cheilitis recurrences [8]. Similar combination has been successfully used in children and adult patients [8, 9]. Camacho et al pointed out the effectiveness of a single injection of triamcinolone immediately after reduction cheiloplasty associated with gradually decreasing doses of tetracycline over a period of 6 months in 27 adult cases [10].

## Conclusion

The association betamethasone and doxycycline is a very interesting alternative therapy for unaesthetic, displaying and resistant cheilitis.
